# Supplementary material for: Photoactivatable Blue Fluorescent Protein
Source: ACS Omega. 2024 Jun 14;9(26):28577–82. doi: 10.1021/acsomega.4c02603 (PMC11223193; doi:10.1021/acsomega.4c02603)
Supplement: Supplementary file 1 — ao4c02603_si_001.pdf [file ao4c02603_si_001.pdf]

SUPPORTING INFORMATION

Title:

Photoactivatable Blue Fluorescent Protein

Authors:

Paul Gaytán* and Abigail Roldán-Salgado

Instituto de Biotecnología, Universidad Nacional Autónoma de México, Av. Universidad 2001, Col. Chamilpa, Cuernavaca, Morelos 62210, Mexico

e-mail: paul.gaytan@ibt.unam.mx

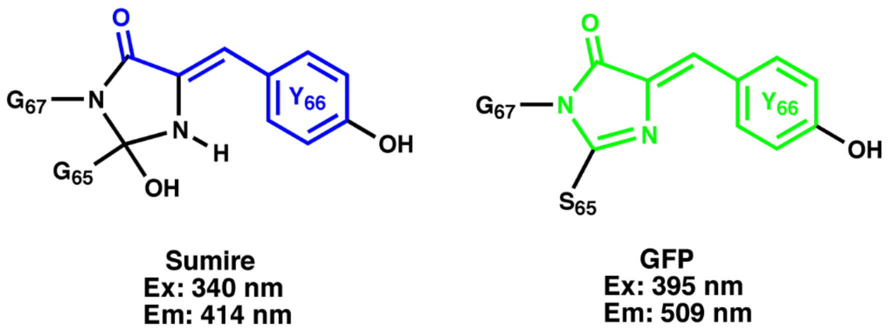

Figure S1. Chromophore structures found in the proteins Sumire and avGFP. Their maximum excitation and emission wavelengths are indicated below the structures. The bonds involved in the system conjugation of π orbitals are shown in blue and green.

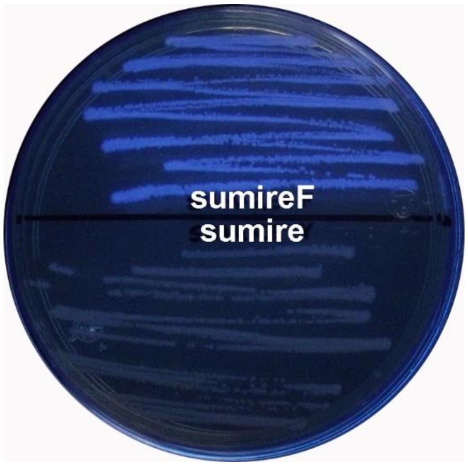

Figure S2. Phenotypical comparison of the expected Sumire protein containing tyrosine at position 165, whereas the fortuitous version contains phenylalanine. This variant was named SumireF. The Petri dish was incubated at 37 °C for 20 h and then refrigerated for 24 h to enhance the maturation of the chromophore in Sumire.

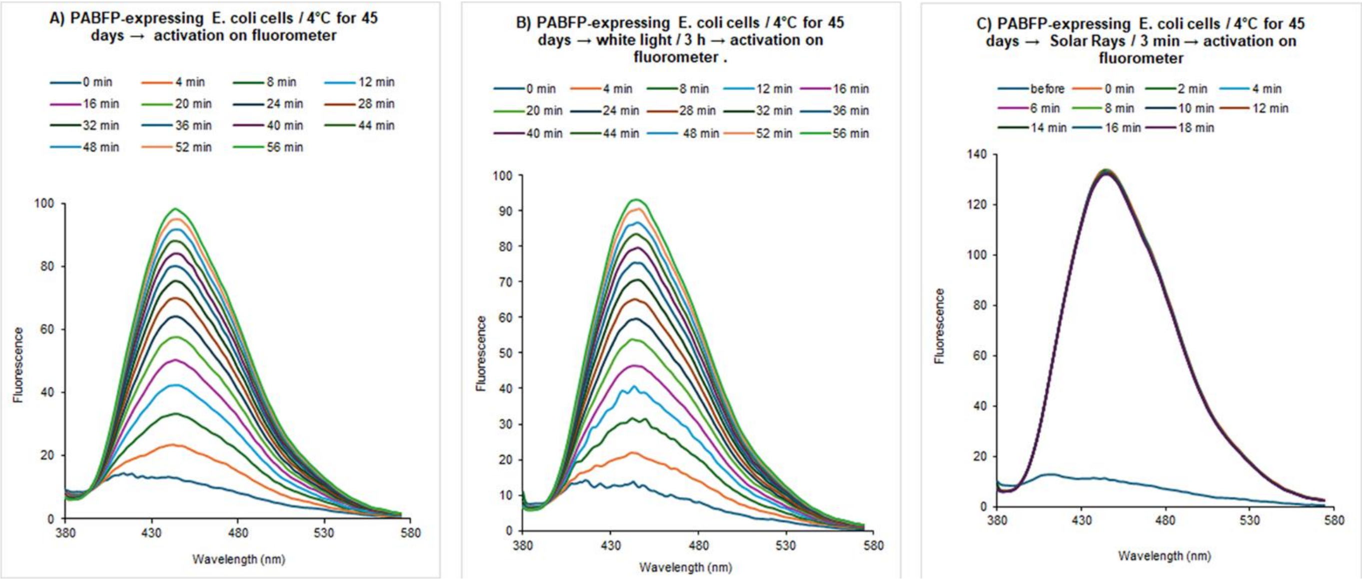

Figure S3. UV activation of *E. coli* cells expressing the dark protein PA-BFP, stored in Petri dishes at 4°C for 45 days in the refrigerator. A) Repetitive acquisition of fluorescence spectra for every four minutes of *E. coli* cells resuspended in PBS buffer. B) Repetition of the experiment shown in A, with a previous exposition of the cell suspension to white light for 3 h. C) Repetition of the experiment shown in A with a previous exposition of the cell suspension to solar rays for 3 min. Excitation: 365 nm; scanning rate: 100 nm/min; excitation slit: 15 nm (maximum allowed); emission slit: 5 nm.

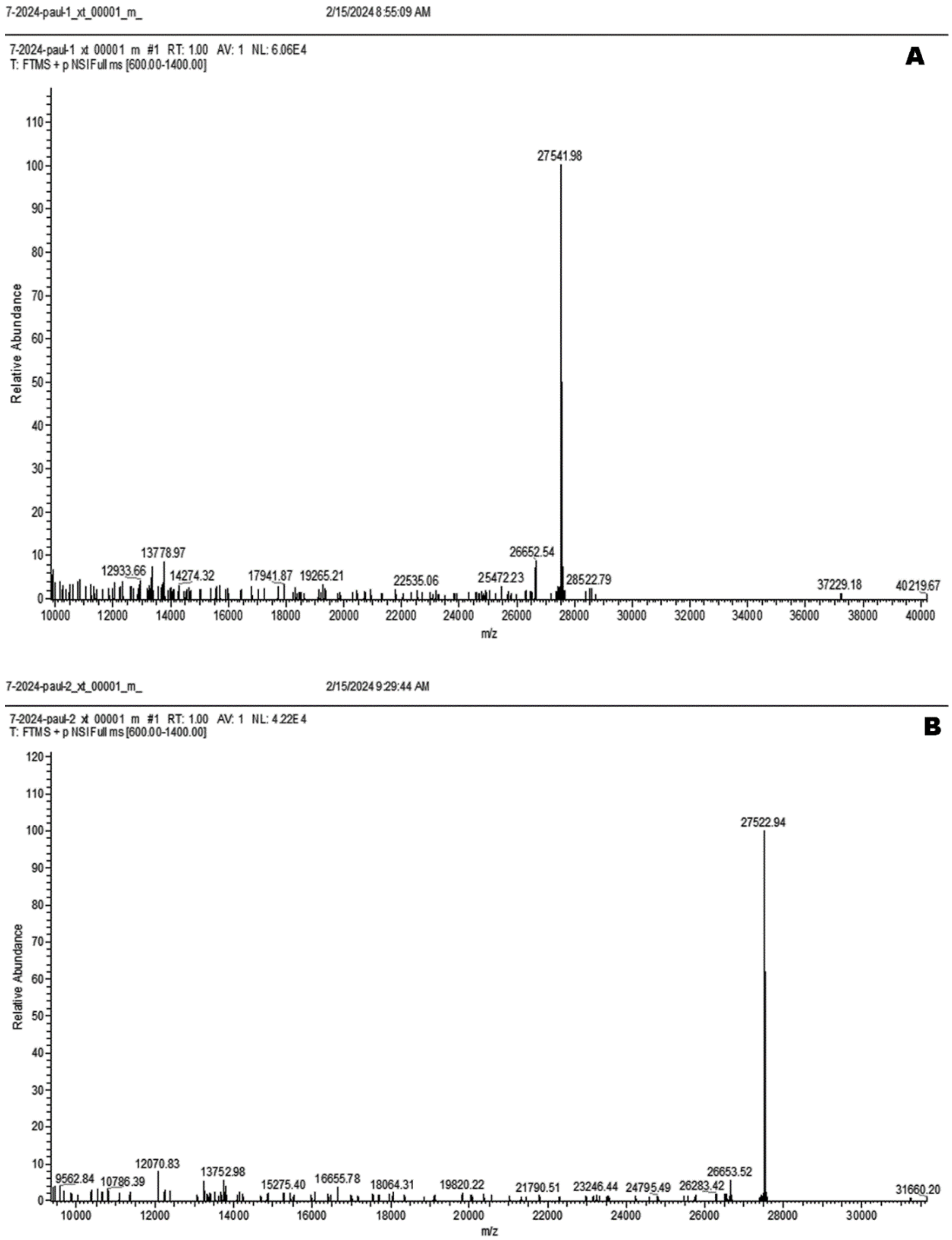

Figure S4. Electrospray ionization mass spectrometry–liquid chromatography of the pure “dark” intermediate photoactivatable blue fluorescent protein (A), and the UV-activated protein (B).

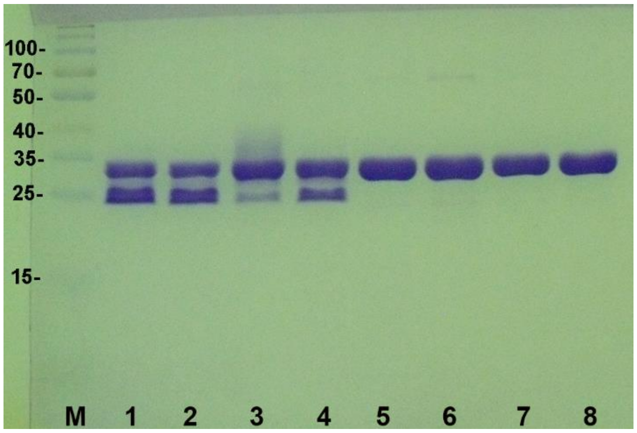

Figure S5. SDS-PAGE of pure proteins. Lanes 1–4: Samples analyzed under semi-denaturing conditions (mixed with denaturing loading buffer, without boiling). Lanes 5–8: Samples analyzed under denaturing conditions (mixed with denaturing loading buffer and boiled). M: Protein marker in KDa. Lanes 1 and 5: sfGFP; lanes 2 and 6: SumireF; lanes 3 and 7: nonactivated PA-BFP; lanes 4 and 8: UV-activated PA-BFP. Analyzed protein: 5 μg per lane. Gel stained with Coomassie Blue.

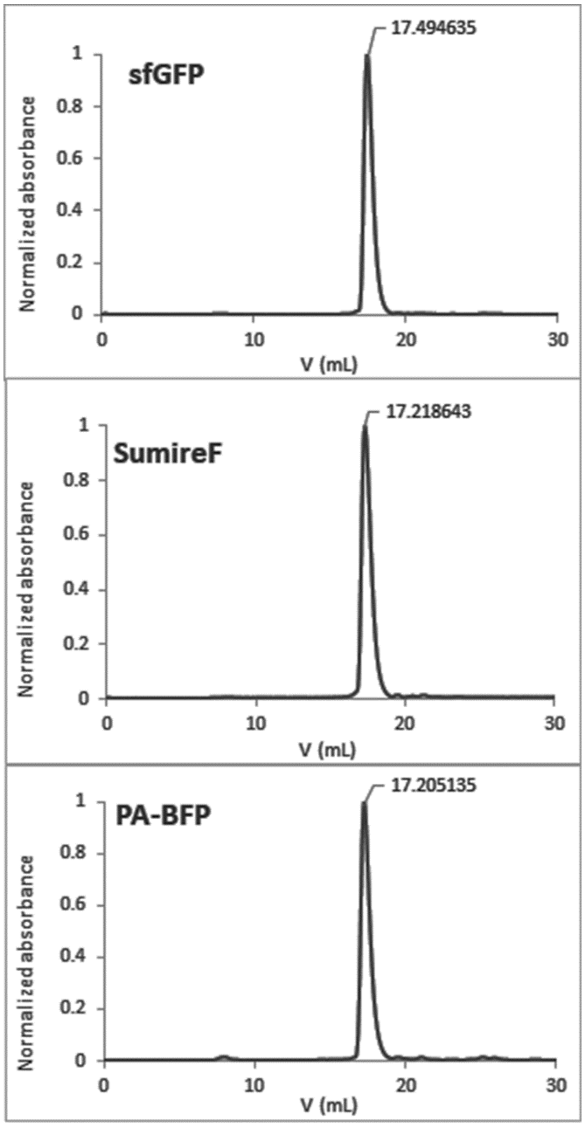

Figure S6. Size exclusion chromatography of the photoactivatable protein PA-BFP and its parental proteins. Column: Superdex™ 200 increase 10/300 GL from GE Healthcare. Buffer: 100 mM phosphate, 0.1 M NaCl. Flow: 0.75 mL/min. The three proteins eluted as monomers.
